# Corneal neurotization for neurotrophic keratopathy: a case report of two patients from different age groups

**DOI:** 10.3389/fopht.2026.1892069

**Published:** 2026-08-25

**Authors:** Hamad M. Alsulaiman, Huda AlGhadeer

**Affiliations:** 1 Oculoplastics and Orbit Division, King Khaled Eye Specialist Hospital & Research Centre, Riyadh, Saudi Arabia; 2 Anterior Segment Division, King Khaled Eye Specialist Hospital & Research Centre, Riyadh, Saudi Arabia

**Keywords:** corneal neurotization, corneal sensation, keratitis, keratopathy, neurotrophic

## Abstract

**Purpose:**

To describe the techniques and the successful outcomes of corneal neurotization (CN) for the treatment of neurotrophic keratopathy (NK) in two patients from different age groups.

**Methods:**

A detailed clinical examination and comprehensive systemic workup were performed to determine the etiology of NK. Both cases were managed surgically with CN and followed for 3 years postoperatively at the King Khaled Eye Specialist Hospital & Research Center, Riyadh, Saudi Arabia.

**Results:**

Two eyes of a 66-year-old male and a 22-month-old boy who were diagnosed with NK and refractory to conservative therapy underwent CN. The first patient underwent indirect corneal neurotization for the management of NK in the right eye from the ipsilateral infraorbital nerve using a sural nerve interposition graft from the left leg, while the second patient underwent indirect corneal neurotization for the management of NK in the left eye from the contralateral right supraorbital nerve using a sural nerve interposition graft from the left leg. The symptoms improved following the procedure as well as the corneal sensation, with improvement of the NK in both patients. At the three-year follow-up, our patients showed no complications or NK recurrence.

**Conclusions:**

CN is a valuable surgical technique for the management of recalcitrant NK and possibly for restoring corneal sensation in some with NK prior to the development of irreversible stromal damage.

## Introduction

Corneal neurotization (CN) is an innovative surgical procedure aimed at restoring corneal trophic and sensory function in patients diagnosed with neurotrophic keratopathy (NK) (1).Traditional therapies largely concentrate on symptom alleviation and complication prevention, although they fail to tackle the nerve dysfunction (2). CN has developed as a viable surgical procedure designed to restore corneal sensation and enhance the ocular surface (2). This approach is employed to manage severe NK, a condition resulting from the impairment of sensory corneal nerves, which leads to recurrent epithelial breakdown and may result in significant consequences such as corneal opacification, ulceration, and perforation (3). Two surgical approaches to CN exist: direct nerve transfer and indirect nerve grafting. In a direct transfer, an intact donor nerve is mobilized, and its distal end is implanted near the recipient cornea. Conversely, indirect neurotization employs a section of a nerve graft that is coapted to a donor nerve at one end and implanted into the cornea at the other end (4–6). This report assesses the long-term outcome of CN performed in our institute in two patients with NK and contrasts the efficacy of indirect neurotization (INT) procedures.

## Case report

### Case 1

**Figure 1:**
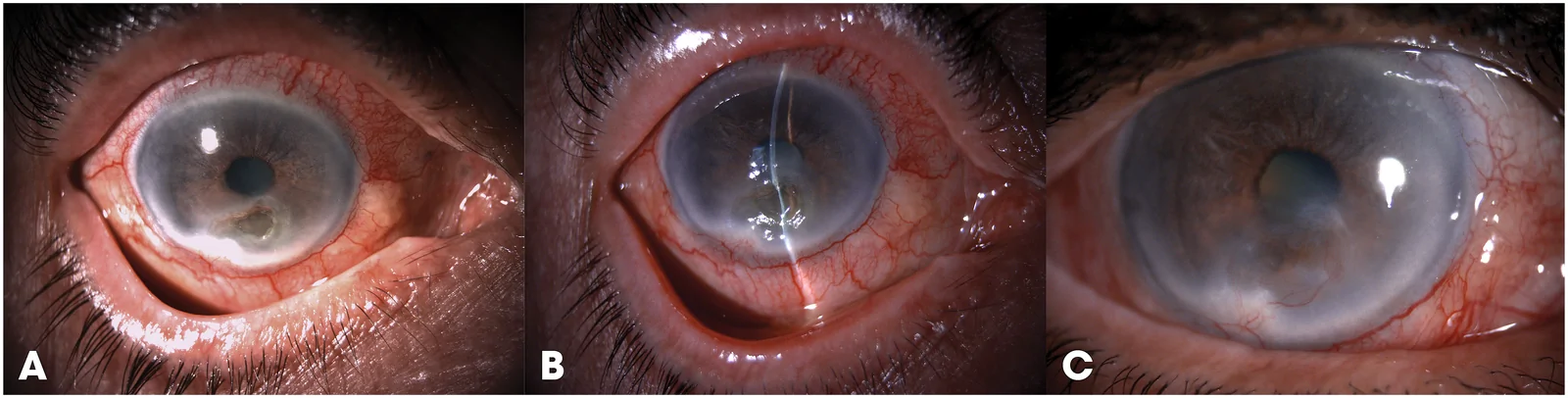
Neurotrophic Keratitis (NK) stage 2 in the right eye post indirect corneal neurotization from the ipsilateral infraorbital nerve using a sural nerve interposition graft from the left leg. **(A)** before treatment. **(B)** Four months after treatment. **(C)** Three years post-surgery (CN).

A 66-year-old male presented to the emergency department of our institute complaining of pain and redness in the right eye. Seven years before his current visit, he underwent an uncomplicated cataract extraction in his left eye. On initial examination, the best-corrected visual acuity (BCVA) was 20/125 in the right eye and 20/300 in the left eye. Intraocular pressure was within normal limit. Slit-lamp biomicroscopy of the right eye revealed an inferior corneal scar, previously recorded, accompanied by an overlying epithelial defect and stromal thinning NK stage 2 (**Figure 1A**). A cataract was also noted in the right eye. Examination of the left eye demonstrated an old inferior corneal scar and a posterior chamber intraocular lens (PCIOL) in place. The patient was initially managed conservatively for the epithelial defect with a bandage contact lens, autologous serum tears, and topical moxifloxacin 0.5% eye drops every 6 hours for two weeks. Due to inadequate clinical improvement, the patient later had an amniotic membrane transplantation in the right eye. The follow-up examination indicated that the epithelial defect had healed; nonetheless, corneal thinning persisted. Corneal sensation was absent upon testing with a cotton wisp (0/4), confirming corneal anesthesia. Sensation in the remaining distributions of the trigeminal nerve remained intact. Given the clinical picture, a presumptive diagnosis of herpetic NK was made. The patient commenced on a therapeutic course of oral valacyclovir 1 g TID for seven days, followed by a prophylactic maintenance dose (500 mg BID) for 3 months. Due to the persistence of corneal anesthesia and thinning, the decision was made to proceed with corneal neurotization. The patient underwent indirect corneal neurotization utilizing a sural nerve interposition graft harvested from the left lower extremity. The graft was coapted end-to-side to the ipsilateral infraorbital nerve. The indirect approach was used because of its technical simplicity, particularly in contralateral innervation. The graft length was established using 4–0 silk of a predetermined length, which was threaded through the tunnel; the graft was first sutured to the host tissue using 8-0 prolene, then sutured to the sclera with 7-0 vicryl. It was subsequently segmented into three fascicles and allocated across the cornea, spanning 6 clock hours from the entrance point. In the immediate postoperative period, the patient was given lubricating eye drops every hour in combination with topical moxifloxacin 0.5% eye drops every 6 hours for two weeks. The early postoperative course was uneventful. Four months postoperatively (**Figure 1B**) BCVA in the affected eye improved to 20/40 and the patient demonstrated recovery of corneal sensation via cotton wisp testing. Prophylactic valacyclovir was discontinued eight months postoperatively. The patient remained stable with no recurrence of the epithelial defect for 3 years (**Figure 1C**).

### Case 2

**Figure 2:**
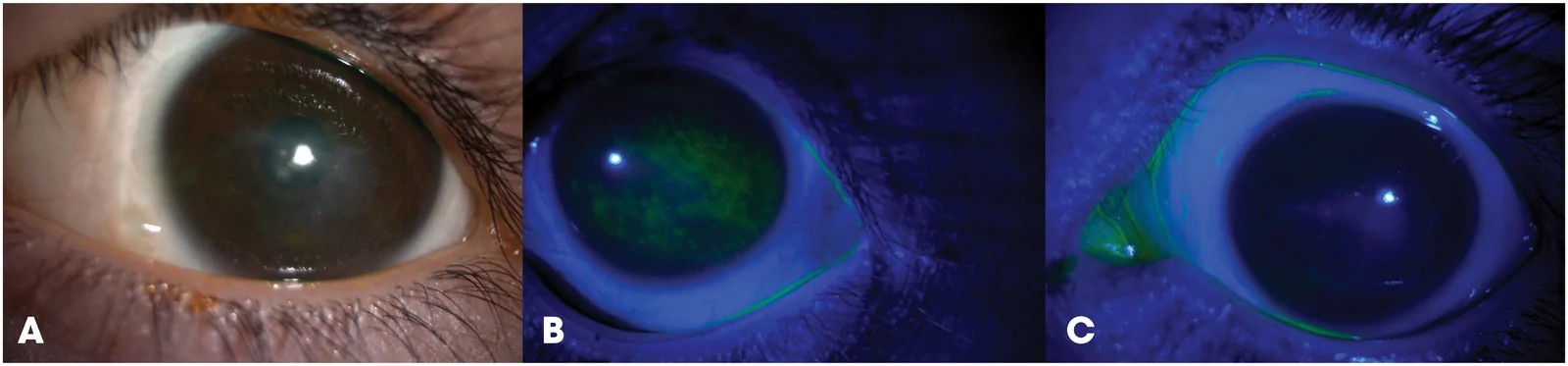
Neurotrophic Keratitis (NK) stage 2 in the left eye post indirect corneal neurotization from the contralateral (right) supraorbital nerve using a sural nerve interposition graft from the left leg. **(A)** before treatment. **(B)** before treatment with fluorescein staining. **(C)** Three years post-surgery (CN).

A 22-month-old boy was referred to our institute for the management of chronic microbial keratitis of the left eye, which had proven refractory to conventional medical therapy. Upon initial examination, the left eye demonstrated a complete absence of corneal sensation. Slit-lamp biomicroscopy revealed a mildly injected conjunctiva and a 4x2 mm corneal infiltrate with an overlying epithelial defect NK stage 2 and inferior corneal neovascularization (**Figures 2A, B**). The anterior chamber was quiet, without evidence of hypopyon; however, the posterior segment could not be visualized. B-scan ultrasonography confirmed a clear vitreous and a flat retina. The patient was initially managed with topical moxifloxacin 0.5% eye drops every 6 hours and ganciclovir gel five times daily. On the eleventh day of admission, the infiltrate showed signs of slow clinical improvement. The decision was made to perform repeat corneal scraping for culture and to initiate oral acyclovir (200 mg). By the nineteenth day of treatment, the corneal epithelial defect had healed completely. The polymerase chain reaction (PCR) test yielded negative results for herpes simplex virus (HSV) and cytomegalovirus (CMV).A comprehensive neurological evaluation revealed a complete absence of sensation across the (V1, V2, V3) of the left trigeminal nerve dermatomal distributions on the left side of the face. Magnetic resonance imaging (MRI) subsequently confirmed the absence of the cisternal and cavernous segments of the left trigeminal nerve, supporting a diagnosis of congenital trigeminal anesthesia. Due to the severity of the neurotrophic state and the risk of recurrent keratitis, the patient underwent corneal neurotization. A sural nerve interposition graft from the left leg was utilized with end-to-end coaptation to the contralateral (right) supraorbital nerve. The same surgical procedure was carried out as in case 1.The postoperative course was uneventful. Over a 3-year follow-up period, while corneal sensation was not recovered, there was no recurrence of corneal epithelial breakdown or microbial keratitis (**Figure 2C**). Furthermore, the residual corneal opacity showed gradual improvement over time.

## Discussion

The cornea is the most sensitive organ in the human body, basically attributed to the ophthalmic branch of the trigeminal nerve, which establishes a dense network of nerve fibers throughout the corneal surface. Corneal innervation is crucial for preserving ocular surface homeostasis. It plays a vital function in the blinking and lacrimal reflexes and facilitates the release of numerous trophic factors essential for epithelial cell proliferation. Hence, any modification in corneal innervation results in epithelial degradation and the onset of NK (2). Previously, intervention was confined to supportive strategies aimed at facilitating epithelial repair (4).

The predominant etiology of NK is herpetic keratitis (27%), followed by systemic disorders, chemical and thermal injuries to the ocular surface, prolonged contact lens usage, excessive application of local anesthetics, central impairment of the trigeminal nerve (such as acoustic neuroma and neurological surgeries), or damage to the ciliary nerves during surgical procedures on the anterior and posterior segments of the eye (7, 8). In our first case, NK resulted from herpetic keratitis, whereas in the second case, NK was attributed to congenital trigeminal hypoplasia.

Corneal sensation assessment is a crucial diagnostic test for NK. Cochet-Bonnet esthesiometry

Provides a quantitative measurement of the corneal sensation compared with the use of a cotton wisp test (8).

The most common surgical procedures include partial tarsorrhaphy, occlusion of the ocular aperture with the aid of application of botulotoxin into the levator palpebrae superioris muscle, conjunctival flap, or transplantation of an amniotic membrane (9). In our patients, conservative management and the available surgical methods fail to restore the corneal sensation; thus, the therapeutic option of corneal neurotization was chosen.

In CN, the preferred choice is often the supraorbital nerve; however, the supratrochlear nerve may also be used, and in rare cases, the infraorbital nerve (10). An ipsilateral approach is indicated only in cases when denervation is confined to the eyeball, specifically affecting only the long ciliary nerves. In some cases, a contralateral technique is used, necessitating the creation of a subcutaneous tunnel to the upper eyelid of the affected eye (11). In indirect neurotization, the supraorbital, supratrochlear or infraorbital is identified. A graft from the sensory nerve, often the sural nerve is sutured to the truncated nerve using end-to-end or end-to-side anastomoses. The nerve graft is inserted into the upper or lower fornix of the affected eye; the exposed nerve fascicles of the graft are passed via tunnels in the subtenon space into the individual quadrants and secured to the sclera in the perilimbal region. Indirect neurotization may use both ipsilateral and contralateral techniques. The disadvantage of this technique is extended operational length, prolonged corneal reinnervation time, and the establishment of a further sensory deficit in the innervation area of the nerve used for the graft (2, 4, 9).

In their initial description of the CN technique, Terzis et al. (12) presented interesting findings from a study involving 6 patients who underwent direct CN utilizing contralateral supraorbital and supratrochlear nerves for unilateral facial palsy and NK, achieving a 100% improvement in corneal sensitivity, visual acuity, and corneal integrity (12). A study conducted by Robbins et al. (13) presents a large retrospective analysis including 58 eyes from 58 patients who underwent CN. Their study indicated that CN is a promising surgical procedure for NK, with significant improvements in NK stage and corneal sensation observed at both the 6–12 month and the final postoperative assessments, which had a median follow-up of 30 months. They conclude that CN is safe and provides effective long-term therapeutic outcomes (13). Rourke et al. described an indirect CN performed on a 74-year-old female with NK, using an indirect CN technique and a sural nerve interposition graft to reinnervate the affected cornea. Six months postoperatively, the patient had marked improvement in their corneal sensation (14). A study from the Czech Republic reported the use of indirect neurotization of the cornea in a 22-year-old patient with a severe form of NK with corneal ulceration and a persisting epithelial defect. The technique of indirect re-innervation of the cornea was used, with a graft of the sural nerve. The corneal findings demonstrated significant improvement post-procedure, with the initial signs of re-innervation observed 5 months after surgery, accompanied by healing of chronic corneal ulceration, and 11 months after the procedure, the cornea is entirely transparent with the exception of a vascularized cornea in the temporal periphery (9). We assessed the long-term outcome of CN in the treatment of refractory unilateral NK in two patients from distinct age demographics. Our findings indicate that CN is a viable, long-term therapy for NK by facilitating the growth of corneal nerves, which stabilize the ocular surface and subsequently reduce the recurrence of NK in the long-term prognosis. The corneal findings in the patients described in our case report significantly improved after the procedure, with healing of chronic corneal ulceration three years following the procedure. We did not encounter any complications or recurrence of NK in our patients. The procedure’s technical complexity, high cost, and multidisciplinary requirements are significant limitations to the widespread use of this advanced technique. In the future, using an esthesiometer to monitor or objectively assess corneal neurotization through *in vivo* confocal corneal imaging would be beneficial.

## Conclusion

Corneal neurotization has the potential to enhance long-term prognosis in patients with NK. Our cases demonstrate improvements of NK. Future studies are required to validate their long-term outcomes over time.

## Data Availability

The original contributions presented in the study are included in the article/supplementary material. Further inquiries can be directed to the corresponding author.
